# Chronic Pancreatitis: One Patient, Multiple Etiologies

**DOI:** 10.7759/cureus.77947

**Published:** 2025-01-24

**Authors:** João Filipe Félix Vieira Afonso, Mafalda Maria Santos, Joana Vieira, Gonçalo Durão-Carvalho, Ana Filipa Rodrigues

**Affiliations:** 1 Internal Medicine, Unidade Local de Saúde do Oeste - Caldas da Rainha, Caldas da Rainha, PRT

**Keywords:** acute on chronic pancreatitis, alcoholic pancreatitis, genetic pancreatitis, pancreatitis causes, treatment of chronic pancreatitis

## Abstract

Pancreatitis, by definition, is an acute inflammation of the pancreas. Acute and chronic pancreatitis can be seen as a spectrum of the same disease rather than two different entities. Here, the authors report the diagnostic and therapeutic approach to a case of a 44-year-old male patient with multiple episodes of acute pancreatitis, leading to the development of chronic pancreatitis. An extended study revealed multiple causes for chronic pancreatitis, such as tobacco, alcohol, and genetics. Pain management and risk factor control were challenging in this case.

## Introduction

Chronic pancreatitis is characterized by inflammation and fibrosis of the pancreatic tissue with loss of acinar and islet cells. In retrospective studies of patients with chronic pancreatitis, approximately 70% were found to have had at least one episode of acute pancreatitis preceding their diagnosis [1]. The incidence and prevalence of chronic pancreatitis are approximately 5-10/100,000 and 120/100,000 people, respectively [2]. It has multiple etiologies. Nevertheless, most patients have more than one etiology such as alcohol (the main cause of chronic pancreatitis in Europe [3,4]), tobacco (responsible for 25% of the cases, having a synergistic relationship with alcohol [3]), hypertriglyceridemia [4], diabetes mellitus [5], hypercalcemia, idiopathic, genetics (mutations in the PRSS1, SPINK1, CFTR, and CTRC genes [6]), autoimmune (Type 1 autoimmune pancreatitis, associated with immunoglobulin G4 (IgG4)-related disease, and Type 2 autoimmune pancreatitis [7]), recurrent and severe acute pancreatitis (after an episode of acute pancreatitis, approximately 10 progress to chronic pancreatitis [4]), and obstructive causes.

The clinical presentation of chronic pancreatitis is characterized by abdominal pain (the most common symptom), typically in the epigastric region radiating to the back. It is primarily caused by alterations in pain signaling of nociceptive neurons [8]. Nausea, vomiting, anorexia, and steatorrhea are also some of the symptoms [9]. Rarely, patients can be asymptomatic. Laboratory findings include elevated amylase and lipase during acute episodes (greater than three times the upper limit of normal, with peak levels decreasing with each additional pain flare); bilirubin and alkaline phosphatase elevation (suggesting compression of the intrapancreatic portion of the bile duct); deficiency of fat-soluble vitamins; elevated triglycerides (in those with chronic pancreatitis due to hypertriglyceridemia); and increased IgG4 in type 1 autoimmune pancreatitis [10]. Imaging modalities include plain abdominal radiographs showing pancreatic calcifications in advanced cases; abdominal ultrasound (increased echogenicity and atrophy [11]); and cross-sectional imaging (CT scans and magnetic resonance cholangiopancreatography (MRCP), which are the best initial diagnostic tests and can establish the diagnosis. In early chronic pancreatitis though, they may not be conclusive; and a direct pancreatic function test with secretin or endoscopic ultrasound (EUS) (for patients with suspected chronic pancreatitis but with CT or MRCP without a clear image [11]) is needed.

Management of chronic pancreatitis involves general measures such as cessation of alcohol and tobacco (to delay disease progression and relieve pain); diet with low-fat meals and high-protein foods; prokinetic medications (for gastroparesis); and vitamin supplementation [12]. Pain management is needed by most patients and nonsteroidal anti-inflammatory drugs (NSAIDs) and acetaminophen are the first line of treatment, while opioids should only be used when the first-line treatment fails [13]. Usually, they are combined with tricyclic antidepressants, selective serotonin reuptake inhibitors, and gabapentinoids to minimize the use of opioid use [13]. For refractory pain, invasive approaches such as celiac plexus block, surgical resection, or endoscopic drainage can be used [13]. In case of exocrine insufficiency, pancreatic enzyme replacement therapy should be initiated [14], whilst in endocrine insufficiency metformin is the preferred oral hypoglycemic agent [15].

## Case presentation

A 44-year-old man presented to the emergency department with postprandial abdominal pain radiating in a band-like pattern to the back, accompanied by nausea and vomiting. He reported a weight loss of 15 kilograms over six months. He denied fever, changes in bowel habits (constipation or diarrhea), steatorrhea, melena, and rectal bleeding. He didn't report any previous infections. On an important note, the patient had been hospitalized three times in the same year for acute alithiasic pancreatitis and had abstained from alcohol since his initial diagnosis. However, he remained an active smoker. His medical history included chronic obstructive pulmonary disease due to heavy smoking (about 38 packs/year), chronic gastritis, and hypertension. His family history revealed that his father also had multiple episodes of pancreatitis. 

On admission, his vital signs were blood pressure 139/94 mmHg, heart rate 67 beats per minute, temperature 36.1ºC, and SpO2 98% (room air). He was sarcopenic with a painful abdomen, especially in the epigastric and right flank regions. A palpable mass of 3-4 centimeters was noted in the epigastric region. Bowel sounds were absent. He was hospitalized for pain management and intravenous fluid therapy due to his inability to tolerate oral intake. Notable laboratory results (Table 1) were similar to previous hospitalizations, including elevated amylase and lipase and normal range values for triglycerides, hepatic function, IgG, and IgG4.

**Table 1:** Laboratory results CRP: C-reactive protein; ESR: erythrocyte sedimentation rate; AST: aspartate aminotransferase; ALT: alanine transaminases; GGT: gamma-glutamyl transferase; LDH: lactate dehydrogenase; IgG: immunoglobulin G; IgG4: immunoglobulin G4

Parameter	Patient Value	Normal Range
White Blood Count	12.6 x 10^3^ u/L	4.0-10.0 X10^3^ u/L
Hemoglobin	12.1 g/L	13.6-18.0 g/L
CRP	6.6 mg/dL	<0.5 mg/dL
ESR	80 mm/hour	12-14 mm/hour
Total Bilirubin	0.5 mg/dL	0.2-1.2 mg/dL
AST	12 U/L	5-34 U/L
ALT	9 U/L	0-55 U/L
GGT	140 U/L	12-64 U/L
Alkaline Phosphatase	72 U/L	40-150 U/L
LDH	185 U/L	125-220 U/L
Amylase	1173 U/L	28-100 U/L
Lipase	4363 U/L	8-78 U/L
Calcium	9.1 mg/dL	8.4-10.2 mg/dL
Glucose	93 mg/dL	70-105 mg/dL
Creatinine	0.63 mg/dL	0.7-1.3 mg/dL
Urea	22 mg/dL	19-44 mg/dL
Triglycerides	187 mg/dL	<150 mg/dL
IgG	742 mg/dL	540-1822 mg/dL
IgG4	70 mg/dL	3.9-86.4 mg/dL

**Figure 1:**
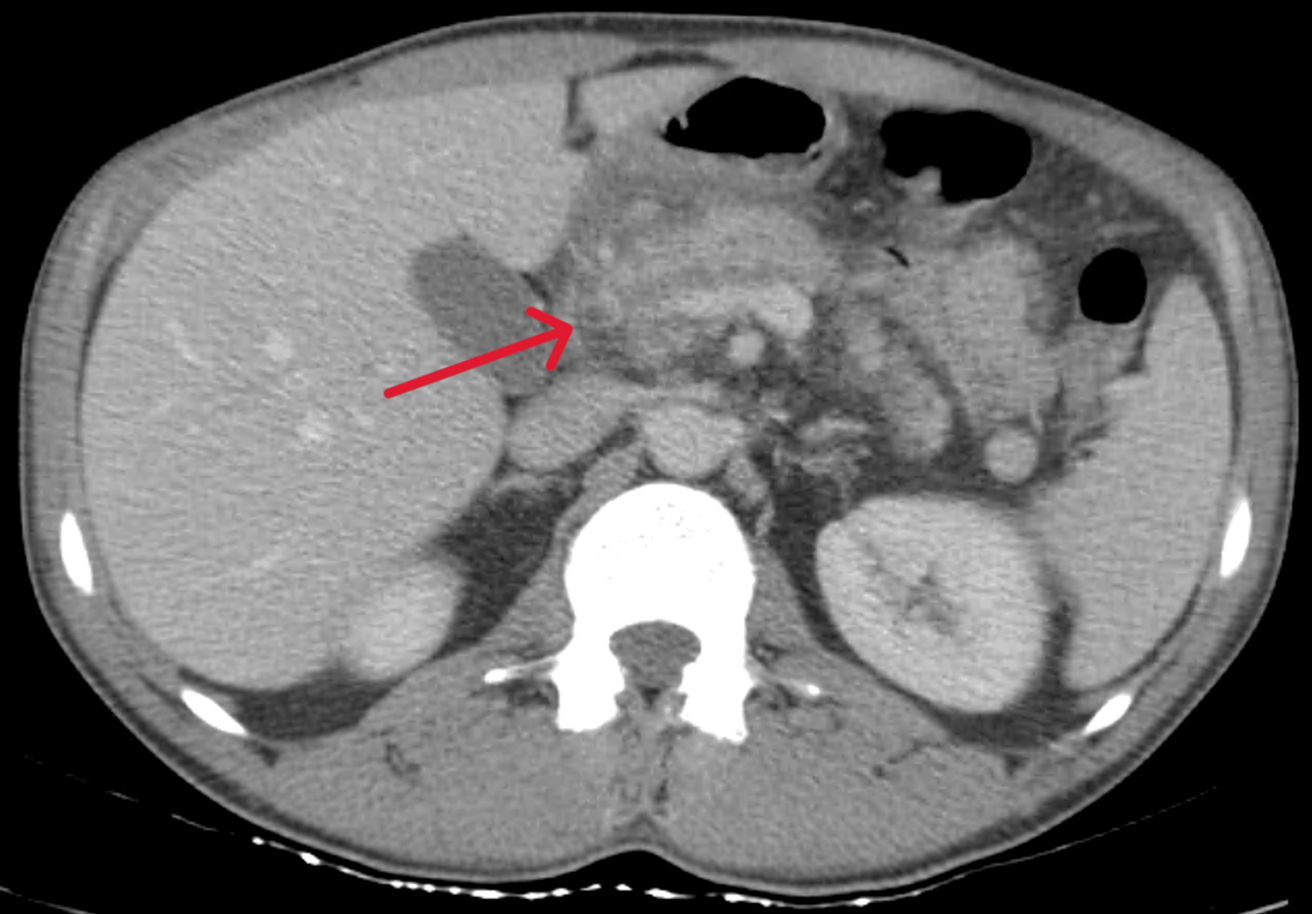
Abdominal CT scan The red arrow signals the diffuse swelling of the pancreas head CT: Computed tomography

**Figure 2:**
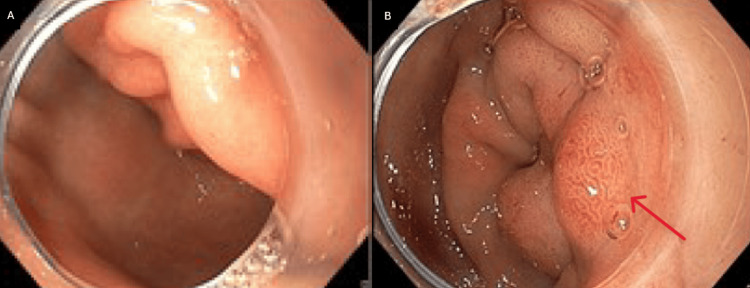
Upper digestive endoscopy: (A) Gastric antrum with edema and (B) duodenal bulb with the red arrow showing the edema and hyperemic regions

**Figure 3:**
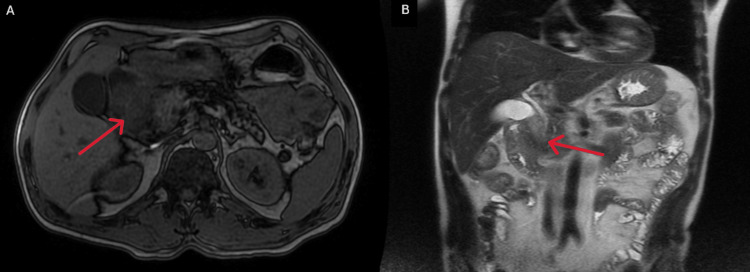
MRCP: (A) T1 axial view and (B) T2 coronal view with the red arrow showing the pancreatic edema compatible with acute pancreatitis MRCP: Magnetic resonance cholangiopancreatography

**Figure 4:**
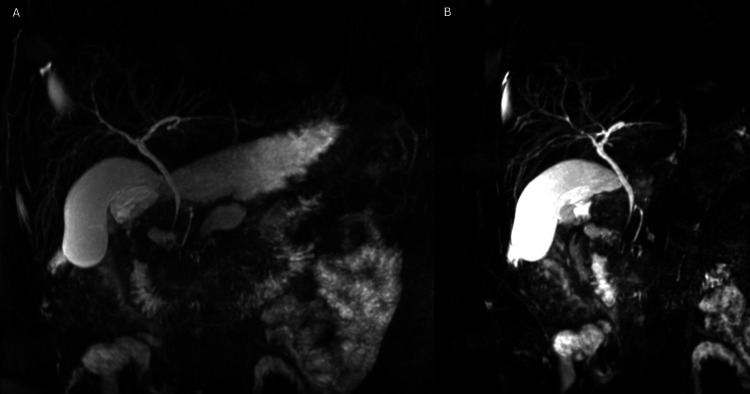
MRCP: No evidence of chronic pancreatitis and biliary or pancreatic neoplasm was found in A and B MRCP: Magnetic resonance cholangiopancreatography

**Figure 5:**
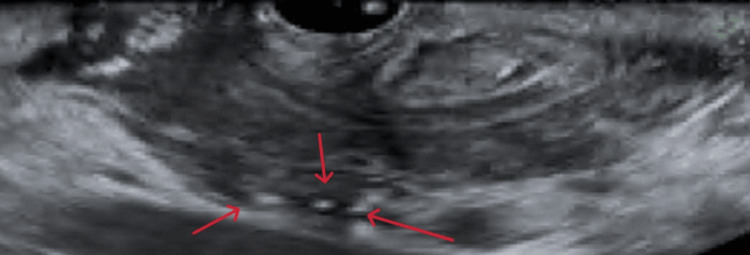
EUS showing multiple calcifications in the pancreas (red arrow) EUS: Endoscopic ultrasound

**Figure 6:**
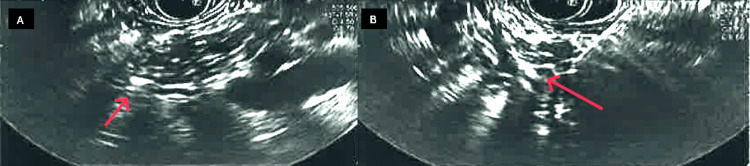
EUS images The red arrows reveal hyperechoic strands suggestive of chronic pancreatitis EUS: Endoscopic ultrasound

An abdominal-pelvic CT scan revealed diffuse pancreatic swelling, particularly in the head, with heterogeneity, ductal ectasia, and parenchymal hypodensity, consistent with acute edematous pancreatitis (Figure 1). Upper digestive endoscopy showed gastrobulbitis, in probable relation with pancreatitis (Figures 2A, 2B). MRCP demonstrated acute pancreatitis without any evidence of biliary or pancreatic neoplasia (Figures 3A, 3B, 4A, 4B). EUS revealed pancreatic heterogeneity with multiple calcifications, consistent with chronic pancreatitis (Figures 5, 6A, 6B). Biopsies showed a moderate lymphoplasmacytic infiltrate with eosinophils, rare positive IgG4 cells, and no signs of malignancy. Genetic testing identified a pathogenic CFTR mutation (c3154T>G, p. (Phe1052Val)) in heterozygosity, associated with autosomal dominant inheritance.

As in previous hospitalizations, it was difficult to control the abdominal pain and the urge to smoke. Pain control was achieved with opioids (tramadol 50 mg every six hours) and adjuvants (gabapentin 100 mg three times a day, amitriptyline 25 mg once a day, and enzymatic supplementation). Despite active collaboration with psychology and psychiatry during hospital stay, the patient did not quit smoking. At follow-up, two months later, the patient reported an improvement in abdominal pain, absence of nausea or vomiting, and an increase in weight.

## Discussion

Chronic pancreatitis is characterized by inflammation, fibrosis, and loss of pancreatic function. There are many causes of chronic pancreatitis, and it usually is due to more than one. The pathophysiology of chronic pancreatitis is not completely understood. It is theorized that there is a common pathway of an initial insult with injury, followed by an attempt at healing through fibrosis and regeneration. Ultimately, in every cause of chronic pancreatitis, there is loss of acinar, islet, and ductal cells, with fibrosis and loss of pancreatic function. Our patient had various possible etiologies for the development of chronic pancreatitis: tobacco, genetic mutation of CFTR, and recurrent acute pancreatitis.

Alcohol is the most common cause. However, less than 5% of heavy alcohol users develop chronic pancreatitis, suggesting that there are other important cofactors that have a say in its development. Tobacco is thought to have a synergistic relationship with alcohol, and it is also by itself an important factor for pain relapse [4]. Genetic mutations are associated with the development of chronic pancreatitis. CFTR is an ion channel that conducts chloride and bicarbonate ions across epithelial cell membranes. Mutations in this gene are responsible for cystic fibrosis, by decreasing the ion channel function and provoking changes in epithelial fluid transport in the lung and pancreas. Heterozygous carriers of CFTR mutations do not develop cystic fibrosis but have an increased risk for pancreatitis. Studies showed that other causes of pancreatitis such as alcohol and tobacco inhibit CFTR function and that in pancreatitis the levels of CFTR expression and its function are diminished. Due to that previously exposed, it is suggested that impairment of CFTR is critical in the development of pancreatitis [6]. Our patient had a pathogenic mutation in the CFTR gene that may have played an important role in the development of the disorder. Recurrent and severe acute pancreatitis is one of the most important risk factors for progression to chronic pancreatitis and, in this case, may have also contributed to the chronicity of the disease [16].

The clinical presentation and laboratory changes of chronic pancreatitis are very similar to the ones that can be found in acute pancreatitis: abdominal pain in the epigastric region with radiation to the back, nausea, vomiting, and anorexia. CT scans and MRCP establish the diagnosis; however, in early stages, the changes may not be visible, and EUS is another alternative. In our patient, CT scans and MRCP only showed signs of acute pancreatitis, while EUS confirmed the presence of chronic pancreatitis.

Autoimmune pancreatitis was also thought to be one of the possible causes. Type 1 autoimmune pancreatitis is associated with IgG4-related disease, where usually, serum levels of IgG4 are elevated at least two times over the upper limit of normal and histology can reveal characteristic findings such as lymphoplasmacytic sclerosing pancreatitis or >10 IgG4-positive cells with periductal lymphoplasmacytic infiltrate, obliterative phlebitis, and/or swirling collagen fibers. Type 2 autoimmune pancreatitis is not associated with an infiltration of IgG4-positive or with elevations in serum levels of IgG4 [7]. However, the histologic diagnostic criteria established by the International Consensus Diagnostic Criteria were not met [17]. Despite this, there are several cases reported of IgG4-negative chronic pancreatitis, without changes in histologic criteria, which responded to corticosteroid therapy [18].

Cessation of alcohol and tobacco and an adapted diet are the pillars of chronic pancreatitis management. Our patient stopped drinking but maintained his smoking habits, despite psychological intervention. Most patients require pain management with analgesics. In our case, NSAIDs and acetaminophen were not effective in pain control, so opioids at a low dose (tramadol) were started in combination with a tricyclic antidepressant (amitriptyline), gabapentin, and pancreatic enzyme supplementation. With this therapeutical strategy, pain control was achieved. Most patients should be referred to a pain specialist. If pain is refractory to the medical approach, surgical options can be used [12].

## Conclusions

Chronic pancreatitis is a complex condition resulting from a combination of multiple etiologies, such as tobacco, alcohol, prior episodes of acute pancreatitis, and genetic predisposition. Symptom management, particularly abdominal pain, remains a challenge for clinicians and often requires assessment from chronic pain specialists, due to its effect on quality of life. Although a patient-centered approach is recommended, abstinence from alcohol and smoking, analgesia, and nutritional guidance remain crucial for reducing inflammation and preventing recurrent episodes of pancreatitis. 
